# Effect of dementia on the incidence, short-term outcomes, and resource utilization of invasive mechanical ventilation in the elderly: a nationwide population-based study

**DOI:** 10.1186/s13054-019-2580-9

**Published:** 2019-08-30

**Authors:** Carmen Bouza, Gonzalo Martínez-Alés, Teresa López-Cuadrado

**Affiliations:** 1 Health Technology Assessment Agency, Carlos III Health Institute, c/Monforte de Lemos 5, Pab 4, 28029 Madrid, Spain; 2 Department of Epidemiology, Columbia University Mailman School of Public Health, New York, NY USA; 3 School of Medicine, Autonomous University of Madrid, Madrid, Spain; 4 National Centre of Epidemiology, Carlos III Health Institute, Madrid, Spain

**Keywords:** Dementia, Elderly, Mechanical ventilation, Incidence, Outcomes, Trends

## Abstract

**Background:**

Though the prevalence of dementia among hospitalized patients is increasing, there is limited population data in Europe about the use of life-support measures such as invasive mechanical ventilation in these patients. Our objective is to assess whether dementia influences the incidence, outcomes, and hospital resource use in elderly patients undergoing mechanical ventilation.

**Methods:**

Using ICD-9-CM codes, all hospitalizations involving invasive mechanical ventilation in adults aged ≥ 65 years were identified in the Spanish national hospital discharge database covering the period 2000–2013. The cases identified were stratified into two cohorts (patients with or without dementia) in which main outcome measures were compared. The impact of dementia on in-hospital mortality and hospital resource use were assessed through multivariable models. Trends were assessed through joinpoint regression analysis and results expressed as average annual percentage change.

**Results:**

Of the 259,623 cases identified, 5770 (2.2%) had been assigned codes for dementia. Cases with dementia were older, had a lower Charlson comorbidity score, and less frequently received prolonged mechanical ventilation or were assigned a surgical DRG. Circulatory disease was the most common main diagnosis in both cohorts. No significant impact of dementia was observed on in-hospital mortality (adjusted OR 1.04, [95% CI] 0.98, 1.09). In the cohort with dementia, the incidence of mechanical ventilation underwent an average annual increase over time of 5.39% (95% CI 4.0, 6.7) while this rate was 1.62% (95% CI 0.9, 2.4) in cases without dementia. However, unlike this cohort, mortality in cases with dementia did not significantly decline over time. Geometric mean hospital cost and stay were lower among cases with than without dementia (− 14% [95% CI − 12%, − 15%] and − 12% [95% CI, − 9%, − 14%], respectively), and these differences increased over time.

**Conclusion:**

This nationwide population-based study suggests no impact of dementia on in-hospital mortality in elderly patients undergoing invasive mechanical ventilation. However, dementia is significantly associated with shorter stay and hospital costs. Our data also identifies a recent marked increase in the use of this life-support measure in elderly patients with dementia and that this increase is much greater than that observed in elderly individuals without dementia.

**Electronic supplementary material:**

The online version of this article (10.1186/s13054-019-2580-9) contains supplementary material, which is available to authorized users.

## Background

Dementia is one of the greatest health problems among persons aged 65 years or older worldwide. Because of population aging and a lack of effective prevention and treatment measures, it has been estimated that in the foreseeable future, the number of affected persons will double [1, 2].

The prevalence of dementia among hospitalized patients is also increasing [3–5], but the balance of potential benefits and harm of intensive care interventions in this population is unclear. In fact, the last few years have witnessed intense debate regarding life-support intensive treatment in patients with dementia such as invasive mechanical ventilation (MV) [6–9]. MV is a key component of the management of critically ill patients with acute or chronic respiratory failure. However, it is associated with a high mortality [10], with short- and long-term complications [11, 12], and requires a complex care level with a substantial impact on hospital resources [13].

Despite these considerations, few studies have examined trends in its use in patients with dementia. The scarce data available come from the USA and Canada where several authors have reported a sustained increased use of MV in patients with dementia in the past few decades [14, 15] with clinical outcomes comparable to those observed in patients without dementia [16].

Spain is a rapidly aging country and dementia prevalences are among the highest in the world (https://www.alzheimer-europe.org/Policy-in-Practice2/Country-comparisons/2013-The-prevalence-of-dementia-in-Europe/Spain). This determines the need to characterize the use of MV and its trends in these patients since the use of effective and safe therapeutic technologies and the appropriate use of healthcare resources are priority objectives in a quality health system. Accordingly, this study sought to examine the characteristics of MV and its recent trends in incidence, associated mortality, and hospital resource use in patients ≥ 65 years with and without dementia based on data from Spain’s national hospital discharge database.

## Methods

### Study design and data sources

We performed a retrospective population-based study using the Spanish Health Ministry’s National Minimum Basic Data Set (MBDS). This official database gathers information derived from discharge reports from all acute-care hospitals in Spain. For each hospitalization, demographic and clinical information is provided including a main diagnosis, 13 secondary diagnoses, and up to 20 procedures coded at each center before patient discharge according to International Classification of Diseases, Ninth Edition, Clinical Modification (ICD-9-CM) codes as well as corresponding diagnosis-related group (DRG) codes. This information, whose registration is mandatory by law in the National Health System, is considered to be representative of the national population as the database covers over 90% of all annual hospital admissions produced in our country [17].

To calculate incidence rates, we used population data provided by the Spanish Statistics Institute [18]. Hospital admissions data were provided by the Ministry of Health, Resources and Welfare [17]. All data used are anonymous so, according to Spanish law, the need was waived for informed consent [19].

### Study population: case definitions and identification

Hospitalizations involving subjects ≥ 65 years who received MV from January 1, 2000, to December 31, 2013, were identified using the ICD-9-CM codes: 96.70 (continuous invasive MV of unspecified duration), 96.71 (continuous invasive MV < 96 consecutive hours) and 96.72 (continuous invasive MV ≥ 96 consecutive hours). These codes are considered specific, stable, and valid [20].

According to ICD-9-CM coding norms, one of these codes is assigned to hospitalized patients who require MV except if used during a routine surgical procedure. Postsurgery MV is coded if lasting more than 2 days or if the clinician reports its duration was longer than planned. MV duration was measured from the moment of endotracheal intubation [17].

Dementia is a clinical syndrome characterized by a global, progressive cognitive impairment that generates functional decline and disability [21]. A variety of brain-damaging conditions, such as degenerative, vascular, metabolic, or toxic brain disease, can lead to dementia, and although the most frequent cause of dementia from middle age to elderly is Alzheimer’s disease, most patients suffer from a mixture of different pathologies [21]. We defined dementia as the presence in any of the diagnoses entered in the database of the ICD-9-CM codes: 290.0–290.9 (dementias), 291.1 (alcohol-induced persisting amnestic disorder), 291.2 (alcohol-induced persisting dementia), 292.82 (drug-induced persisting dementia), 292.83 (drug-induced persisting amnestic disorder), 294.0 (amnestic disorder in conditions classified elsewhere), 294.1 (dementia in conditions classified elsewhere), 294.2 (dementia, unspecified), 294.8 (other persistent mental disorders due to conditions classified elsewhere), 294.9 (unspecified persistent mental disorders due to conditions classified elsewhere), 331.0 (Alzheimer’s disease), 331.1 (frontotemporal dementia), 331.2 (senile degeneration of the brain), 331.7 (cerebral degeneration in diseases classified elsewhere), 331.82 (dementia with Lewy bodies), and 331.9 (cerebral degeneration, unspecified). Similar broad definitions of dementia have been used by others [14] to improve case detection.

To assess the comorbidity burden, we used the Charlson index score validated by Deyo [22] and improved for ICD-9-CM [23] according to secondary diagnoses. For the purposes of this study, dementia itself was excluded in the Charlson Index [24].

For every case, the main diagnostic group was assessed according to the ICD-9-CM chapters: infectious disease (001–139), neoplasms (140–239), endocrine diseases (240–279), hematological diseases (280–289), neurological diseases (320–389), diseases of the circulatory system (390–459), respiratory diseases (460–519), diseases of the digestive system (520–579), diseases of the genitourinary tract (580–629), diseases of the skin and subcutaneous tissue (680–709), diseases of the musculoskeletal system and connective tissue (713–739), and injury-poisoning (800–999).

### Data analysis

We conducted a descriptive and comparative analysis of cases with and without dementia, including demographic and clinical information, comorbidity burden, and hospital mortality, stay, and costs. Charlson comorbidity scores are provided as a continuous variable and as categorical with 4 groups (0, 1–2, 3–4, > 4) of increasing severity and impact on outcomes [25]. Categorical variables are expressed as absolute frequencies and percentages while continuous variables are given as geometric means and geometric standard deviation (SD), as geometric means are less influenced by extreme values than arithmetic ones. To test differences in categorical variables, we used Pearson’s chi-square test.

In-hospital mortality was estimated as the number of deaths relative to the number of cases and expressed as a percentage, or case fatality rate (CFR). To examine the effect of dementia on mortality, stay, and costs, we conducted regression models (logistic regressions for mortality and linear regressions of log-transformed stay and costs) and adjusted them in two multivariable models. Model 1 was adjusted for baseline characteristics (age, sex, Charlson index) whereas model 2 also included the principal diagnosis at admission and MV duration. Results are expressed as odds ratios (OR) with 95% confidence intervals (for mortality) and geometric means ratio with 95% confidence intervals (for stays and costs).

We examined temporal trends in MV incidence rate (per 100,000 people and per 10,000 hospital discharges), proportion of MV cases with dementia, prolonged MV (defined as a duration of ≥ 96 consecutive hours according to the ICD-9-CM codification system), and CFR. To this end, we used joinpoint regression models—generalized linear models that assume a Poisson distribution [26]. In these models, any apparent trend can be statistically assessed through a Monte Carlo permutation method [27]. Trends are presented as the average annual percentage change (AAPC), a summary measure of the overall trend over the study period. To compare the AAPC of both study groups, we examined whether their regression mean functions were parallel, allowing for different intercepts, using the Pairwise comparison parallel test. In addition, we analyzed trends in length of stay and costs using Cuzick’s *p*-trend test.

All tests were performed using the packages STATA 15 (StataCorp. LP, College Station, TX, USA) and Joinpoint Regression 4.7.0.0. Significance was set at *p* < 0.05.

## Results

Out of the 19,979,322 hospitalizations in persons aged ≥ 65 years produced over the 14-year study period, 259,623 cases underwent MV. Of these, 5770 (2.2%) were cases with dementia.

As may be seen in Table 1, in the dementia cohort, the proportions of women and older age strata were higher than in the cohort without dementia. The Charlson score, however, was lower indicating a lower comorbidity burden among those with dementia. Among the main comorbidities, we should highlight a greater presence of cerebrovascular disease in the group of patients with dementia. Circulatory disease was the most common main diagnosis in both cohorts and was followed with disparate frequency in each one by respiratory disease, injury-poisoning, and digestive disorders. Conversely, cancer was much less frequent in the cases with dementia. Registries including dementia corresponded more to smaller hospitals, and patients less frequently received prolonged MV or were assigned a surgical DRG than in the non-dementia group. The cohort with dementia showed a slightly, yet significantly, higher in-hospital mortality, and this difference was attributable only to cases subjected to short-duration MV (Additional file 1: Table S1). However, as may be observed in Table 2, according to the logistic regression analysis adjusted for age, sex, comorbidity burden, main diagnosis, and MV duration, dementia had no significant impact on in-hospital mortality.

**Table 1:** General characteristics of adults ≥ 65 years receiving invasive mechanical ventilation

	With dementia	Without dementia	OR (95%CI)	*p* value
	5770 (2.2)	253,853 (97.8)		
Gender women	2702 (46.8)	96,946 (38.2)	1.43 (1.35, 1.50)	< 0.001
Age				
65–74 years	1787 (31.0)	131,717 (51.9)	Ref.	
75–84 years	3166 (54.9)	108,990 (42.9)	2.14 (2.02, 2.27)	< 0.001
> 84 years	817 (14.1)	13,146 (5.2)	4.58 (4.21, 4.99)	< 0.001
Charlson Index score				
0 points	1891 (32.8)	77,478 (30.5)	Ref.	
1–2 points	2932 (50.8)	124,467 (49.0)	0.97 (.91, 1.02)	0.235
3–4 points	734 (12.7)	37,678 (14.8)	0.80 (0.73, 0.87)	< 0.001
> 4 points	213 (3.7)	14,230 (5.6)	0.61 (0.53, 0.71)	< 0.001
Main Charlson comorbidities				
Diabetes	1476 (25.6)	59,879 (23.6)	1.10 (1.03, 1.14)	< 0.001
Cerebrovascular disease	1048 (18.2)	18,972 (7.5)	2.75 (2.57, 2.94)	< 0.001
COPD	1005 (17.4)	54,918 (21.6)	0.76 (0.71, 0.82)	< 0.001
Heart failure	836 (14.5)	47,921 (18.9)	0.73 (0.68, 0.78)	< 0.001
ICD-9-CM main diagnosis				
Circulatory	1751 (30.3)	98,683 (38.9)	0.69 (0.65, 0.73)	< 0.001
Respiratory	984 (17.0)	39,379 (15.5)	1.12 (1.04, 1.20)	0.001
Injury-poisoning	910 (15.8)	27,338 (10.8)	1.55 (1.44, 1.67)	< 0.001
Digestive	805 (14.0)	29,621 (11.7)	1.23 (1.14, 1.32)	< 0.001
Cancer	364 (6.3)	29,229 (11.5)	0.52 (0.47, 0.58)	< 0.001
No. of hospital beds				
< 200	691 (12.0)	26,929 (10.6)	Ref	
200–500	1927 (33.4)	71,698 (28.2)	1.05 (0.96, 1.14)	0.302
501–1000	1774 (30.8)	87,898 (34.6)	0.79 (0.72, 0.86)	< 0.001
> 1000	1378 (23.9)	67,327 (26.5)	0.80 (0.73, 0.87)	< 0.001
DRG surgical	2455 (42.6)	145,001 (57.2)	0.56 (0.53, 0.59)	< 0.001
Mechanical ventilation ≥ 96 h	1729 (30.0)	85,162 (33.6)	0.85 (0.80, 0.90)	< 0.001
In-hospital mortality (CFR)	2992 (51.9)	123,445 (48.6)	1.14 (1.08, 1.20)	< 0.001

Data presented as number of cases (%)

*OR* odds ratio, *CI* confidence interval, *COPD* chronic obstructive pulmonary disease, *DRG* diagnosis-related group, *CRF* case fatality rate

**Table 2:** Impact of dementia on in-hospital mortality and hospital resource use

	With dementia	Without dementia	Crude	Model 1	Model 2
	(%)	(%)	OR (95% CI)	Adjusted OR (95% CI)	
CFR	51.9	48.6	1.14 (1.08, 1.20)	1.05 (0.99, 1.1)	1.04 (0.98, 1.09)
	Geometric mean (SD)	Geometric mean (SD)	Ratio of geometric means (95% CI)	Adjusted ratio of geometric means (95% CI)	
Hospital stay, days	11.16 (3.20)	14.01 (3.19)	0.80 (0.77, 0.82)	0.85 (0.82, 0.87)	0.88 (0.86, 0.91)
Costs, €	10,423 (2.34)	12,855 (2.40)	0.81 (0.79, 0.83)	0.83 (0.81, 0.85)	0.86 (0.85, 0.88)

Model 1: adjusted for sex, age, Charlson index

Model 2: adjusted for sex, age, Charlson index, main diagnosis, and length of MV

*CFR* case fatality rate, SD standard deviation

Among the survivors, 78% of cases with dementia and 81% of those without dementia were discharged home while 16.3% and 15.1% respectively were discharged to long-term care centers.

Both geometric mean stay and hospital costs for the dementia group were significantly lower than for the non-dementia group (Table 2). Further, multivariate analysis of the impacts of dementia on hospital resource use indicated that dementia was associated significantly with a shorter adjusted mean hospital stay and lower mean hospital costs per case.

### Temporal trends

**Fig. 1:**
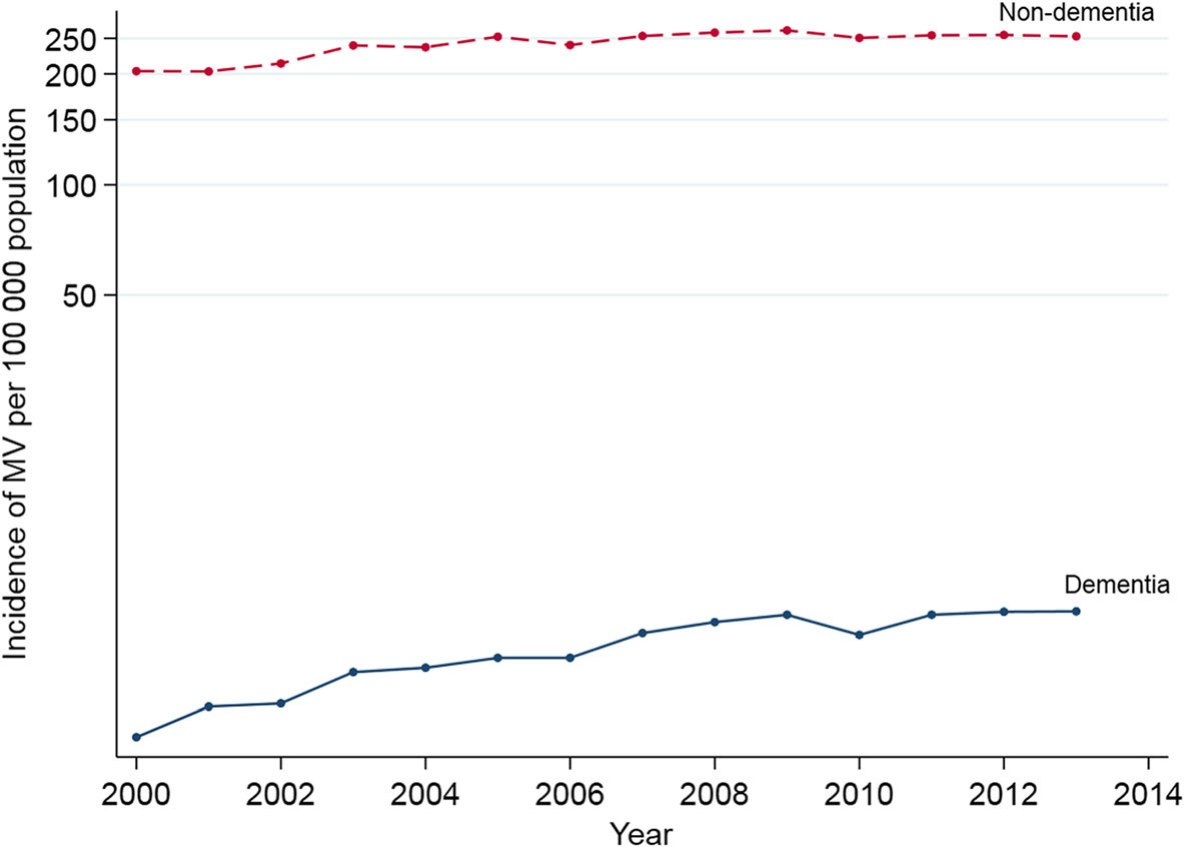
Trends in invasive mechanical ventilation incidence rates in patients with and without dementia. The figure shows the changes observed over time in incidence rates. The cohort with dementia reaches a greater increase than the cohort without dementia

Rates of MV use referred to hospital discharges and the general population underwent a significantly greater increase among the subjects with dementia compared to those without (Table 3). In patients with dementia, the rate of MV use per 10,000 hospital discharges went up from 1.76 in 2000 to 3.57 in 2013, with an AAPC of 4.7%. Meanwhile, in patients without dementia, the rate of MV use per 10,000 hospital discharges went up from 114.5 in 2000 to 131.3 in 2013 with an AAPC of 0.91%. According to the comparability test, trends of MV in dementia and non-dementia cases were different (*p* value for test for parallelism = 0.005). The population incidence of MV in people with dementia rose from 3.1 to 6.9 per 100,000 inhabitants ≥ 65 years, yielding an AAPC of 5.39%, while in the non-dementia group it increased from 203.6 to 253.3, for an AAPC of 1.62% (Fig. 1). According to the comparability test, population incidence trends in dementia and non-dementia were also different (*p* value for test for parallelism = 0.03).

**Table 3:** Trend analysis

	MV in cases with dementia			MV in cases without dementia			Parallelism test
	2000	2013	AAPC (95% CI)	2000	2013	AAPC (95% CI)	
Proportion (%)	1.51	2.64	3.8 (3.0, 4.5)^†^	98.5	97.4	− 0.1 (− 0.1, − 0.1)^†^	*P* < 0.001
Hospital discharge rate (per 10,000)	1.76	3.57	4.71 (3.6, 5.8)^†^	114	131	0.91 (0.4, 1.4)^†^	*P* = 0.005
Population rate (per 100,000)	3.1	6.9	5.39 (4.0, 6.7)^†^	203.6	253.3	1.62 (0.9, 2.4)^†^	*P* = 0.030
MV ≥ 96 h (%)	24.6	28.4	− 0.8 (− 2.3, 0.7)	31.2	34.9	0.99 (0.7, 1.3)^†^	*P* = 0.031
CFR (%)	55.9	50.2	− 0.30 (− 1.1, 0.5)	51.9	44.5	− 1.19 (− 1.3, − 1.0)^†^	*P* = 0.10

*MV* invasive mechanical ventilation, *AAPC* average annual percentage change, *95%CI* 95% confidence interval, *CFR*: case fatality rate

^†^Statistically significant

**Fig. 2:**
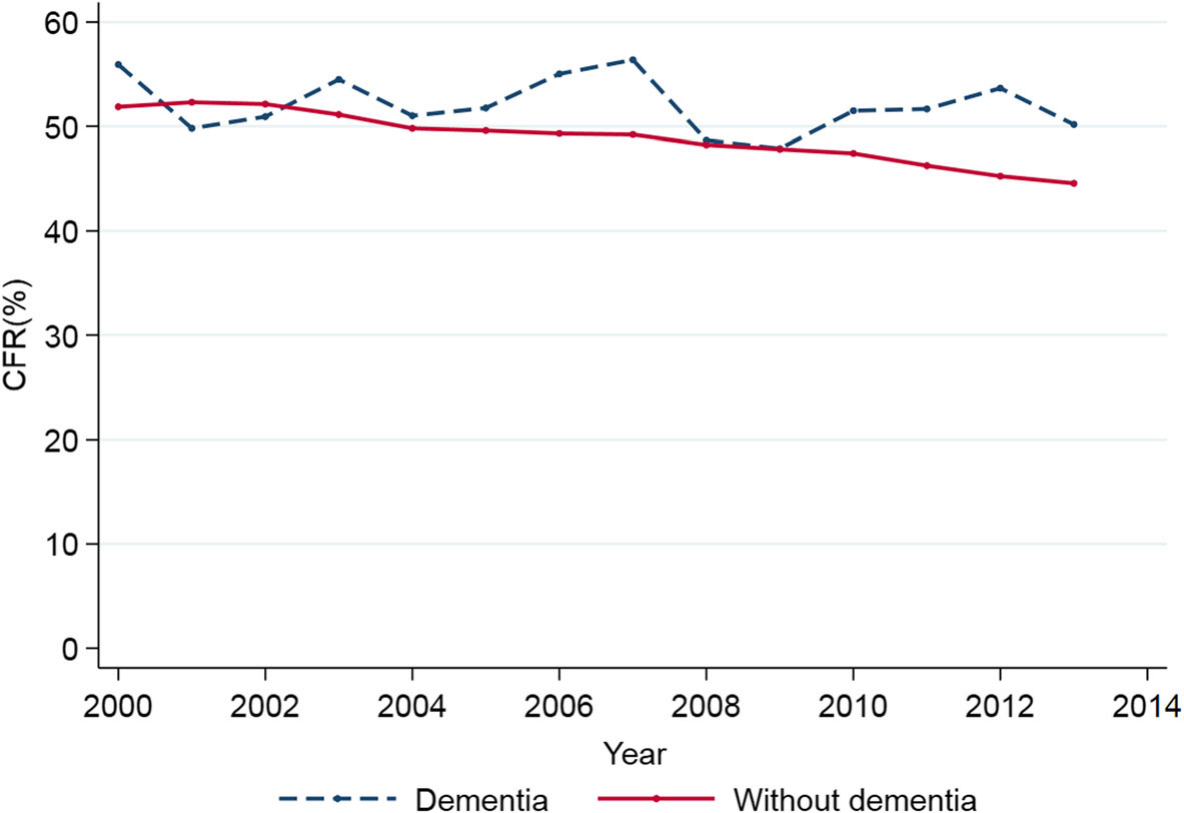
Trends in in-hospital mortality in MV episodes of patients with and without dementia. The figure shows the changes observed over time in in-hospital mortality. Note that cases with dementia do not show the downward trend detected in the cases without dementia; rather, they feature a fluctuating trend over the period of study

Table 3 also provides temporal trends in the use of VM ≥ 96 h and in-hospital mortality (CFR), indicating that subjects with dementia underwent no significant changes over the study period, while in the non-dementia cohort the use of prolonged MV increased and mortality decreased. As shown in Fig. 2, cases with dementia have not followed the descending trend shown by the cases without dementia; rather, mortality has fluctuated over time.

**Fig. 3:**
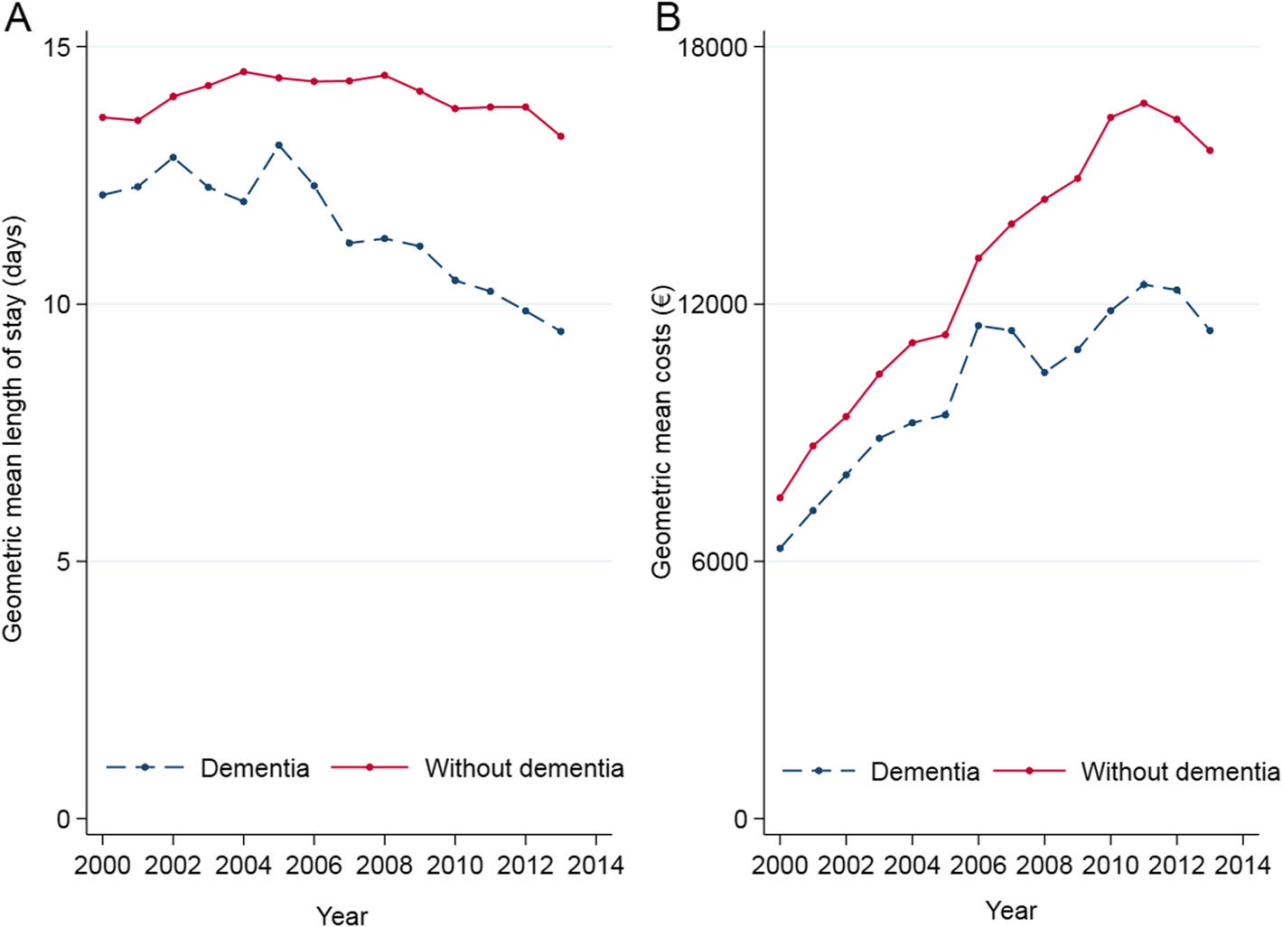
Trends in hospital resources in patients with and without dementia. **a** Over the period examined, geometric mean hospital stay has decreased significantly in cases with dementia passing from 12.12 days in the year 2000 to 9.47 days in 2013. In contrast, in cases without dementia, mean stay has remained stable passing from 13.63 to 13.26. **b** Over the period examined, geometric mean costs per case in the group with dementia have risen significantly from 6305€ in 2000 to 11,384€ in 2013 and in cases without dementia from 7485€ to 15,583€ (both *p* < 0.001)

Figure 3 illustrates that geometric mean hospital stay in the cohort with dementia has steadily declined over time, while it has been relatively stable in the cohort without dementia. Geometric mean hospital costs per case have risen markedly over the study period. This increase has been nevertheless lower among cases with dementia, and inter-cohort differences have persisted (Fig. 3).

## Discussion

The findings of this population-based study indicate that the use of MV in elderly persons diagnosed with dementia has shown a marked increase in Spain between the years 2000 and 2013. Further, this increase has been higher than that observed in their dementia-free counterparts. They also reveal that while no impacts of dementia on hospital mortality were detected, the declining trend in mortality produced in patients without dementia was not observed. In addition, compared with subjects free of dementia, these individuals incur lower hospital costs and length of stay.

As far as we know, this is the first study to characterize the pattern of MV use in adults ≥ 65 years with and without dementia in a European country. The demographic and clinical characteristics of our cases are similar to those described in the USA and Canada [14, 15], although dementia appears as notably less frequent among those receiving MV in our setting. Only 2.2% of hospitalized adults aged ≥ 65 years undergoing MV had been assigned a code for dementia, contrasting with the 15% and the 8.6% rates described in those studies. Our results show in the cohort of patients with dementia a marked increase in the incidence of MV with an average annual increase of 5.39%, which is much higher than the 1.62% observed in the cohort without dementia. Notwithstanding, the increase detected was markedly lower than that reported by Lagu (11.4%) and by Borjaille (7.8%) in adults ≥ 65 years with dementia [14, 15]. In part, these differences could be explained by the very different healthcare systems and the organizational models used for the care of critically ill patients between countries [28]. We should not forget that in a healthcare setting, offer is an important regulator of demand. Effectively in the USA, the increased use of MV in patients with advanced dementia has been linked to a greater availability of beds in intensive care units [29]. In Spain, with its universal, equal-access healthcare system, it is estimated that in 2010 there were some 9.6 ICU beds per 100,000 inhabitants [30, 31] while in the USA this was around four times this figure in 2009 [32]. But, in spite of these differences, our data are in line with those reported in North America and contrast with prior studies indicating that acute care patients with dementia are treated substantially less aggressively than patients without dementia [8].

Our dementia group showed an older age and higher percentage of women, as described by others [16]. However, our cases had a lower comorbidity burden which could be partly due to the different score system used as there is still no standardized method to assess this issue despite its important role in patient’s outcomes [33]. For this study, we selected the Charlson comorbidity index as it has shown a similar capacity to scales based on physiological scores to predict mortality in critically ill patients [34].

As expected, in-hospital mortality was really high in both cohorts. But, a main finding of our study was the lack of significant differences in CFR between both cohorts once adjusted for remaining clinical-demographic variables, meaning that dementia has not had a significant impact on hospital mortality in individuals ≥ 65 years subjected to MV. This finding, which is in line with the data reported by Lagu et al. [16], also suggests the use of MV in persons with dementia in our country complies (at least in terms of mortality as an effect measure) with the quality standard of its use in patients ≥ 65 years without dementia.

Our trends analysis, nevertheless, indicates that while hospital mortality in the dementia-free cohort has steadily declined over the 14 years examined, which is consistent with literature reports [35], we observed no parallel changes in the dementia cohort.

Something similar can be said about the trend observed in the use of hospital resources. While some studies have shown that dementia leads to longer mean hospital stay and costs in older patients admitted because of acute illness [3, 4], our finding is consistent with more recent descriptions [16, 36] that these variables are significantly lower in patients with dementia. Our study also reveals that this reduced resource use in patients with dementia persists when the extent of MV was introduced as a covariate in the adjusted multivariate model. Additionally, trend analysis indicate that mean length of stay difference has increased over the years since while the mean hospital stay in cases without dementia has remained stable, it has progressively decreased in cases with dementia.

Unfortunately, the database design prevents any causal inferences or assessment of other reasons that could justify these trends, such as the existence of advanced directives, family preferences, or clinical practices toward the limitation of therapeutic efforts and the use, instead, of comfort measures. We consider those data are fundamental and that it is necessary to study them in a prospective way.

Our observations extend the scarce available information on the incidence and short-term outcomes of the use of invasive technologies such as MV in adults aged ≥ 65 years with dementia, and perhaps, they can be of help in the existing debate about the use of these therapeutic measures in patients with dementia. Further, given the national population-based nature of our data, we feel they may be generalizable and of interest for clinical decision making and healthcare resource planning in an increasingly aging society for which a greater prevalence of dementia is foreseen in the near future [15, 37, 38].

### Limitations

Our study has several limitations we should mention. When working with clinical-administrative data, sensitivity to detect the variables of interest depends directly on the discharge report completed by the responsible physician. There is evidence to suggest that dementia has been undercoded in discharge reports, especially in mild or complex cases [39]. To minimize this limitation, we used a broad definition of dementia in line with previous, similar studies [14, 16], even though these definitions have not been validated against clinical charts. Moreover, MV is a major procedure which is easily identified in a patient’s clinical record and whose ICD-9-CM codes are stable and validated [20]. However, we did not have access to staging information of our dementia cases. Given that an inverse relationship has been established between dementia severity and the frequency at which patients are hospitalized and that some clinical guidelines emphasize the need to treat persons with dementia at their homes [37], it is likely that our dementia cohort will contain a high proportion of mild-moderate severity cases. Our data source also prevents us from knowing other individual factors such as pharmacological treatments; the existence of an advance directive or their socio-cultural or educational level which makes it impossible to further characterize this cohort and stratify the results according to these factors. Nonetheless, given the universal character of our national health system with equal access to the whole population, aspects such as the socioeconomic level have not influenced the results obtained. Likewise, given the regulation of our national health system, we can assume that clinical and coding practices have not been related to economic incentives. Also, our database does not include physiology-based scores of common use in ICUs, such as APACHE or SAPS. Notwithstanding, Christensen and colleagues have shown that the Charlson comorbidity index performs similarly to physiology-based scores at predicting short- and long-term mortality for ICU patients [34]. Finally, because of this study’s retrospective nature, we cannot rule out that temporal trends may, at least in part, be associated with different treatment practices during the long period of study. However, the population nature of our study, its main strength, means we can assume a lack of selection bias and can also extrapolate its results. In addition, RECORD recommendations for reporting of results were followed [40].

## Conclusions

This nationwide population-based study reveals no impact of dementia on in-hospital mortality in elderly patients undergoing invasive mechanical ventilation. However, dementia is significantly associated with shorter stay and hospital costs. Our data also identifies a recent marked increase in the use of this life-support measure in elderly patients with dementia and that this increase is much greater than that observed in elderly individuals without dementia. This data have important implications for clinical decision-making and healthcare resource planning in an increasingly aging society for which a greater prevalence of dementia is foreseen in the near future.

## Additional file

Additional file 1: **Table S1.** General characteristics and outcomes of cases by duration of invasive mechanical ventilation (MV). (DOCX 16 kb)

## Data Availability

The data are anonymized and, according to Spanish law, are exempt from the necessity for informed consent. They come from hospital discharge records collected and de-identified by the Spanish Ministry of Health, Social Services and Equality. The authors requested and obtained access to the data from the Ministry and, due to a signed confidential agreement, cannot share these data with third parties. However, these records are publicly available for research purposes. Requests of access to the data should be addressed directly to the Ministry.
