# Supplementary material for: Effect of dementia on the incidence, short-term outcomes, and resource utilization of invasive mechanical ventilation in the elderly: a nationwide population-based study
Source: Crit Care. 2019 Aug 30;23:291. doi: 10.1186/s13054-019-2580-9 (PMC6716901; doi:10.1186/s13054-019-2580-9)
Supplement: Supplementary file 1 — Table S1. General characteristics and outcomes of cases by duration of invasive mechanical ventilation (MV). (DOCX 16 kb) [file 13054_2019_2580_MOESM1_ESM.docx]

**Table S1.** General characteristics and outcomes of cases by duration of invasive mechanical ventilation (MV)

|  | **MV≥96h** | |  | **MV<96h** | |  |
| --- | --- | --- | --- | --- | --- | --- |
|  | **With Dementia** | **Without Dementia** | **OR (95%CI)** | **With Dementia** | **Without Demencia** | **OR (95% CI)** |
|  | **N=1729** | **N=85162** |  | **N=4041** | **N=168 691** |  |
| **Gender women** | 42.0 | 36.9 | 1.24 (1.13, 1.37) | 48.9 | 38.9 | 1.51 (1.41,1.60) |
| **Age** |  |  |  |  |  |  |
| 65-74 y | 36.6 | 54.0 | 1 | 28.6 | 50.8 | 1 |
| 75-84 y | 54.8 | 42.3 | 1.91 (1.72, 2.11) | 54.9 | 43.3 | 2.26 (2.10, 2.43) |
| >84 y | 8.6 | 3.7 | 3.42 (2.85, 4.10) | 16.5 | 5.9 | 4.97 (4.51, 5.48) |
| **Charlson** **Index** |  |  |  |  |  |  |
| 0 points | 31.5 | 31.3 | 1 | 33.3 | 30.1 | 1 |
| 1-2 points | 51.5 | 48.2 | 1.06 (0.95, 1.18) | 50.5 | 49.5 | 0.92 (0.86, 0.99) |
| 3-4 points | 13.5 | 14.9 | 0.90 (0.77, 1.05) | 12.4 | 14.8 | 0.75 (0.68, 0.84) |
| **>**4 points | 3.5 | 5.6 | 0.62 (0.47, 0.81) | 3.8 | 5.6 | 0.61 (0.52, 0.72) |
| **Main Comorbidities** |  |  |  |  |  |  |
| Diabetes | 24.3 | 20.0 | 1.28 (1.15, 1.44) | 25.4 | 25.0 | 1.02 (0.95, 1.10) |
| Cerebrovascular disease | 19.3 | 8.6 | 2.56 (2.26, 2.89) | 17.7 | 6.9 | 2.88 (2.65, 3.13) |
| COPD | 18.3 | 21.1 | 0.84 (0.74, 0.95) | 17.0 | 21.9 | 0.73 (0.67, 0.79) |
| Congestive Heart Failure | 15.1 | 19.4 | 0.72 (0.63, 0.83) | 14.2 | 18.5 | 0.73 (0.67, 0.80) |
| **ICD-9-CM main diagnostic** |  |  |  |  |  |  |
| Circulatory | 29.3 | 31.4 | 0.90 (0.81, 1.01) | 30.8 | 42.6 | 0.69 (0.65, 0.72) |
| Respiratory | 19.5 | 18.9 | 1.04 (0.92, 1.17) | 16.0 | 13.8 | 1.12 (0.65, 0.73) |
| Injury-poisoning | 15.4 | 12.9 | 1.24 (1.08,1.41) | 15.9 | 9.7 | 1.76 (1.61, 1.92) |
| Digestive | 13.1 | 12.8 | 1.03 (0.89, 1.18) | 14.3 | 11.1 | 1.34 (1.22, 1.46) |
| Cancer | 5.4 | 10.7 | 0.48 (0.39, 0.59) | 6.7 | 11.9 | 0.53 (0.47, 0.60) |
| **No of hospital beds** |  |  |  |  |  |  |
| <200 | 9.7 | 8.9 | 1 | 12.9 | 11.5 | 1 |
| 200-500 | 33.0 | 27.9 | 1.09 (0.91, 1.29) | 33.6 | 28.4 | 1.05 (0.94, 1.16) |
| 501-1000 | 28.2 | 33.9 | 0.76 (0.64, 0.91) | 31.8 | 35.0 | 0.81 (0.73, 0.89) |
| >1000 | 29.0 | 29.2 | 0.91 (0.76, 1.09) | 21.7 | 25.1 | 0.76 (0.68, 0.85) |
| **DRG surgical** | 52.3 | 64.7 | 0.60 (0.54, 0.66) | 38.4 | 53.4 | 0.54 (0.51, 0.58) |
| **Inhospital mortality** | 53.7 | 54.1 | 0.98 (0.89, 1.08) | 51.1 | 45.9 | 1.23 (1.16, 1.31) |

Data presented as percentage of cases (%); OR odds ratio, CI confidence interval, COPD chronic obstructive pulmonary disease, CHF congestive heart failure, DRG diagnosis related group, CRF case fatality rate, h hours, d days, y years.
